# Haemoglobin Levels in Early Life among Infants with and without Retinopathy of Prematurity

**DOI:** 10.3390/ijerph18137054

**Published:** 2021-07-01

**Authors:** Edwin Pheng, Zi Di Lim, Evelyn Tai Li Min, Hans Van Rostenberghe, Ismail Shatriah

**Affiliations:** 1 Department of Ophthalmology and Visual Science, School of Medical Sciences, Universiti Sains Malaysia, Kubang Kerian 16150, Kelantan, Malaysia; edwinpcm@hotmail.com (E.P.); limzidi@hotmail.com (Z.D.L.); 2 Hospital Universiti Sains Malaysia, Jalan Raja Perempuan Zainab 2, Kota Bharu 16150, Kelantan, Malaysia; hansvro@usm.my; 3 Department of Paediatrics, School of Medical Sciences, Universiti Sains Malaysia, Kubang Kerian 16150, Kelantan, Malaysia

**Keywords:** retinopathy of prematurity, haemoglobin, infant, premature

## Abstract

Retinopathy of prematurity (ROP) is a proliferative retinal vascular disorder attributed to an ischaemic stimulus in preterm infants. Haemoglobin, the main component for oxygen transportation, may be implicated in ROP development. This retrospective study compared the mean weekly haemoglobin levels between infants with and without ROP over the first six weeks of life. Premature infants of less than 32 weeks gestational age and less than 1.5 kg birth weight were grouped into age and birth weight-matched ROP cases and controls. Weekly mean haemoglobin levels were documented. An independent t-test was used to analyze the difference in mean haemoglobin levels between infants with ROP and infants without ROP. Adjustment for confounders was performed using one-way analysis of covariance. There was a statistically significant difference in adjusted mean haemoglobin levels between the ROP and non-ROP group during the first week of life (*p* = 0.038). No significant intergroup differences were observed at the other weeks. Haemoglobin monitoring during the first week of postnatal life may be useful to guide ROP screening in premature infants.

## 1. Introduction

Retinopathy of prematurity (ROP) is a major cause of preventable childhood blindness [1,2,3], characterized by proliferative retinopathy occurring at the junction of the normal vascularized retina and the peripheral avascular retina. It stems from incomplete vascularization of the retina in preterm infants, causing ischemia and consequent abnormal vascularization and fibrosis [4]. Major risk factors for development of ROP include low birth weight, low gestational age, a history of blood transfusion, sepsis, intraventricular haemorrhage, and necrotizing colitis [5,6,7,8,9].

ROP screening is performed in infants with a birth weight of less than 1500 g and gestational age of less than 32 weeks [10,11]. Screening of ROP should be done in a timely manner, as prompt treatment may reduce the risk of long term adverse visual outcomes [3]. However, the benefits of screening need to be balanced against the pharmacological risks of mydriatics and the stress of serial retinal examinations [10]. Despite pre-existing guidelines for ROP screening, the vision-threatening sequelae of this developmental condition still affect a significant number of infants [12,13]. Thus, there remains a need for improved screening criteria for ROP among premature infants.

Studies regarding the role of haemoglobin in ROP have yielded varying conclusions [14,15,16,17,18,19,20]. Most studies on this topic have treated haemoglobin as a categorical variable (i.e., anaemia present vs absent), or only measured haemoglobin at certain time points, with large gaps in between. We believe that this approach runs the risk of missing key points in ROP development. As haemoglobin is a relatively simple parameter to monitor and its testing is universally available, we hope that identifying its role in ROP may enhance our screening programs by allowing earlier detection of ROP. Our study thus aimed to compare the weekly mean haemoglobin levels between premature infants with and without ROP in the first six weeks of life.

## 2. Materials and Methods

### 2.1. Study Population

A retrospective study was performed among premature infants admitted to Hospital Universiti Sains Malaysia from September 2016 to December 2019. Approval was obtained from the Human Research Ethics Committee of Universiti Sains Malaysia (USM/JEPeM/18090441). The tenets of the declaration of Helsinki were adhered to throughout. Premature infants of less than 32 weeks gestational age and less than 1.5 kg birth weight were included. Those with congenital deformities, ocular diseases, or individual/parental history of haemoglobin-related disease (e.g., thalassemia) were excluded. The ROP cases were equally matched with non-ROP controls (1:1) of equal gestational age and birth weight. Extremely small gestational age or low birth weight infants who could not be matched with the control group were excluded.

### 2.2. Data Collection

Birth weight, gestational age, volume of blood transfusion, duration of oxygen supplementation, culture-proven sepsis, intraventricular haemorrhage, enterocolitis, congenital heart disease, pneumonia, gender, ROP stage, requirement for ROP treatment, and weekly mean haemoglobin levels were collected from birth until week six of life. Haemoglobin levels were measured using the Automated Blood Cell Analyzer (Sysmex XN-1000™ Hematology Analyzer), with 500 microLiters of blood per phlebotomy collected in a K_2_EDTA tube. Culture-proven sepsis was defined as one episode of positive blood culture during the first six weeks of life. ROP was staged based on the revised ‘International Classification of Retinopathy of Prematurity’ [21,22]. This classifies ROP into 5 stages; Stage 1—a demarcation line, i.e., a line separating the avascular and vascularised retina; Stage 2—a ridge; Stage 3—extraretinal fibrovascular proliferation; Stage 4—partial retinal detachment, extrafoveal (Stage 4a) or foveal (Stage 4b); and Stage 5—total retinal detachment. Treatment was based on recommendations from the ‘Early Treatment For Retinopathy of Prematurity Randomized Trial’ [23].

### 2.3. Statistical Analysis

Statistical analyses were performed using IBM SPSS Statistics for Windows, Version 26.0 (IBM Corp, Armonk, NY, USA). An independent t-test was used to determine the mean differences in numerical variables between ROP and non-ROP infants. A chi-square test was performed to determine the differences in categorical risk factors between the two groups. An independent t-test was also performed to determine the mean differences in weekly mean haemoglobin levels from birth to week six of life between ROP and non-ROP infants. The mean haemoglobin level was then adjusted for confounding factors using one-way analysis of covariance (ANCOVA). Comparison of mean haemoglobin level between ROP cases requiring treatment and controls was similarly performed.

## 3. Results

A total of 62 premature infants were included (31 in the ROP group and 31 in the control group). The ROP and non-ROP groups did not differ significantly in gestational age (*p* = 0.968), nor in birth weight (*p* = 0.443). Infants with ROP had a significantly higher volume of total blood transfusion, longer days of oxygen supplementation, and a higher proportion of culture-proven sepsis than infants without ROP (*p* < 0.001). The clinical characteristics of all the infants are summarized in Table 1.

There was a statistically significant difference in mean haemoglobin levels between the ROP group and the non-ROP group in the first week of life (*p* = 0.002). This difference persisted even after adjustment for confounders (*p* = 0.038). There were no significant differences in mean haemoglobin level between the two groups at birth, nor from week two until week six of life (Table 2).

There were significant differences in mean haemoglobin levels between ROP cases who required treatment and controls at birth (*p* = 0.33), week 1 (*p* = 0.02), week 3 (*p* = 0.012) and week 4 (*p* = 0.013). These differences were not observed after adjustment for confounders (Table 3).

## 4. Discussion

There are an estimated 15 million babies born prematurely each year worldwide [24,25]. Declines in neonatal mortality and increased survival of extremely preterm infants contribute to increased ROP incidence [4]. As the leading cause of preventable blindness among premature infants, ROP is becoming a public health concern [26,27]. The World Health Organization’s (WHO) “Vision 2020” program has identified retinopathy of prematurity as one of the leading causes of preventable blindness in children [27], underscoring the importance of increasing vigilance for ROP via improved screening guidelines.

Ashton et al. and Patz et al. [28,29] have theorized that the development of ROP is a two-phase process. They postulated that during the first week of life, exposure to atmospheric oxygen results in hyperoxia relative to the intrauterine environment. This causes retinal vessels to undergo vasoconstriction, which may lead to decreased availability of growth factors such as serum insulin-like growth factor 1 (IGF-1), resulting in aberrant vessel development and failure of complete vascularization of the retina. After the first week, relative hypoxia occurs in the face of increased metabolic demands, stimulating increased production of vascular endothelial growth factor. This culminates in the growth of abnormal vessels at the junction between vascularized and avascularized retina. These abnormal vessels are the main culprits that cause the sequelae of ROP. Haemoglobin, with its role in oxygen transport, would appear to be implicated in the development or exacerbation of ROP. However, a literature review on the association of haemoglobin with ROP produced conflicting results (Table 4). Our study clarifies the significance of mean adjusted haemoglobin levels during early life in preterm infants with regard to ROP risk.

We observed that infants in the ROP group had significantly lower mean haemoglobin levels in the first week of life compared to the group without ROP. These differences persisted even after adjusting for confounding factors. Banerjee et al. likewise found an association between low haemoglobin at birth and ROP development [14]. There were no significant differences in adjusted mean haemoglobin levels between infants with ROP requiring treatment and controls. This is in keeping with the results of Lundgren et al. [17]. These findings indicate that lower haemoglobin in the first week of life is associated with ROP, but does not influence its severity.

Haemoglobin levels in a preterm infant depend on physiological and non-physiological factors [32]. The physiological factors include rate of body growth, shortened red blood cell life span, and cardiovascular factors such as decreased erythropoietin production after the switch from placenta-based to lung-based oxygen delivery; while the non-physiological factors include inadequate nutritional intake, non-laboratory blood loss, and sepsis [33,34]. Higher haemoglobin levels in preterm infants are associated with increased blood iron storage [35], improved neural development [36], and lower rates of intraventricular haemorrhage [37]. Conversely, anaemia in preterm infants is associated with slower weight gain, poor oral intake, higher risk of cardiovascular events, and exacerbation of apnoeic episodes [38].

**Figure 1:**
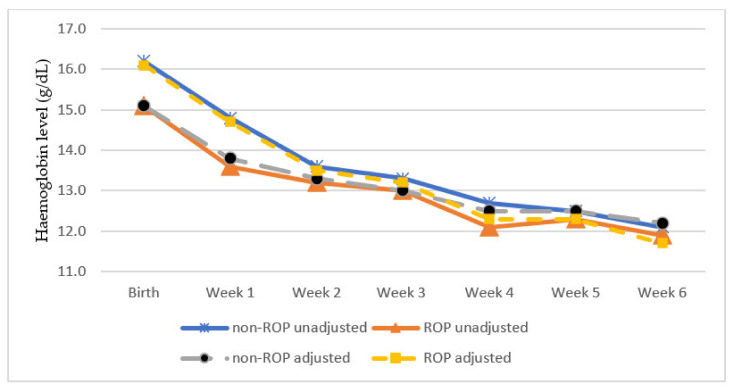
Mean haemoglobin levels in the first 6 weeks of life.

Both groups (with and without ROP) experienced decreases in mean haemoglobin level from birth till the sixth week of life (Figure 1). This is attributed to the immature erythropoietic system of premature infants, termed anaemia of prematurity [39]. However, no significant intergroup differences in adjusted haemoglobin levels were observed after the first week of life. Our study, which has a follow up duration comparable to that of Lundgren et al., thus confirms that within the critical period of ROP development, haemoglobin levels are only significant in the first week of life [17]. Anaemia may exacerbate retinal hypoxia, triggering the proliferative stage of ROP [40]. This effect may be compounded by the presence of blood transfusions, prolonged oxygen supplementation, and sepsis.

Blood transfusion and low haemoglobin are inextricably intertwined in the pathogenesis of ROP [41,42]. Low haemoglobin levels are believed to have a direct hypoxic effect on the retina, while blood transfusion may result in a high hematocrit level and corresponding oxidative stress, leading to vessel damage and inefficient oxygen transport [42]. The role of oxygenation in the development of ROP was hypothesized by Michelson, Ashton et al., and Patz et al., and corroborated by animal studies showing that exposure to high oxygen concentration results in the development of retinal vessels that grow into the vitreous [43,44,45]. Extremely low oxygenation levels are related to death, cerebral palsy, patent ductus arteriosus, pulmonary vascular resistance, and apnoea [46]. Conversely, high levels of oxygenation are related to ROP and chronic lung disease [46].

Haemoglobin in neonates at birth predominantly consists of foetal haemoglobin, which has a higher affinity for oxygen than adult haemoglobin [47]. Blood transfusions may shift the ratio of fetal and adult haemoglobin in these infants, as haemoglobin transfused is almost exclusively of the latter type. A greater proportion of adult haemoglobin may result in increased availability of oxygen to tissues, causing relative hyperoxia, which is believed to preface the hypoxic stage of ROP.

Sepsis has been associated with ROP by various mechanisms [48]. Low birth weight infants have higher susceptibility to infection, which subsequently leads to increased release of proinflammatory cytokines into the circulation [49]. These cytokines, including interleukin-6, interleukin-1-beta, and tumor necrosis factor alpha, inhibit IGF-1 [49,50,51]. IGF-1 functions to stabilize nascent vessels during angiogenesis [52]. Lower levels of IGF-1 have been associated with the development of ROP [53]. Additionally, sepsis-induced dysregulation of erythropoiesis may compound the disruption of normal circulatory and respiratory function in these ill infants, indirectly contributing to ROP development [54]. These processes highlight the need for regulation of oxygen supplementation, control of sepsis, and the avoidance of unnecessary blood transfusions.

Infants with untreated ROP have a wide range of vision outcomes, with no light perception as the worst-case scenario [55]. Even in those with treated ROP, approximately 4.1—30% will have visual impairment due to a variety of causes including refractive errors, amblyopia, strabismus, and retinal detachment [56]. As these children will require long term ophthalmic follow-up and management, it is paramount to identify parameters which can assist in screening infants for ROP. Our findings of lower haemoglobin in early life among infants with ROP may guide decision-making with regard to ROP screening among high-risk premature infants.

To the best of our knowledge, this is the only study focusing on haemoglobin levels in infants with and without ROP that controls for gestational age and birth weight. As haemoglobin levels are lower in more premature infants as well as those with lower birth weight [57], failure to account for these may confound results. Other strengths of our study are its close evaluation of serial weekly mean haemoglobin levels during the critical period of ROP development, and adjustment for multiple confounders [58]. The present study is limited by the nature of its retrospective design. Future directions for research may include the use of other haematological indices to allow earlier detection and treatment of this sight-threatening disease [31]. Besides this, the incorporation of haemoglobin levels into software algorithms, such as WINROP, which automatically alerts the clinician in cases of increased ROP risk, may also be beneficial [58,59,60]. These strategies may permit more efficient and cost-effective ROP screening, which could improve visual outcomes among premature infants.

## 5. Conclusions

Infants with ROP have significantly lower mean haemoglobin levels than infants without ROP during the first week of life. These findings may guide future ROP screening in high-risk infants.

## Figures and Tables

**Table 1:** Comparison of clinical characteristics between ROP and non-ROP group.

Characteristic	ROP *n*= 31	non-ROP *n*= 31	*p*-Value
Birth weight in grams (mean ± SD)	962.2 ± 167.9	960.6 ± 147.8	0.968 †
Gestational age in weeks (mean ± SD)	27.5 ± 1.8	28.0 ± 1.4	0.443 †
Volume of blood transfusion in ml (mean ± SD)	104.2 ± 62	53.9 ± 28	<0.001 *†
Days of oxygen supplementation (mean ± SD)	14.4 ± 17.1	3.1 ± 4.7	0.001 *†
Culture proven sepsis	16 (51%)	1 (3%)	<0.001 *
Intraventricular haemorrhage	17 (54%)	20 (64%)	0.605 ‡
Enterocolitis	5 (16%)	7 (22%)	0.749 ‡
Pneumonia	10 (32%)	8 (26%)	0.780 ‡
Congenital heart disease	10 (32%)	7 (22.5%)	0.570 ‡

† Independent t-test. ‡ Pearson chi-square test. * *p* < 0.05 is statistically significant. SD, standard deviation.

**Table 2:** Comparison of weekly mean haemoglobin between ROP and non-ROP groups.

Mean Haemoglobin (g/dL)				
Period	non-ROP *n*= 31	ROP *n*= 31	*p* Value ^1^	*p* Value ^2^
Birth	16.2 ± 2.6	15.1 ± 1.7	0.057	0.158
Week 1	14.8 ± 1.4	13.6 ± 1.4	0.002 *	0.038 *
Week 2	13.6 ± 1.4	13.2 ± 1.2	0.259	0.496
Week 3	13.3 ± 1.2	13.0 ± 1.3	0.430	0.490
Week 4	12.7 ± 1.1	12.1 ± 0.9	0.036 *	0.185
Week 5	12.5 ± 0.9	12.3 ± 1.4	0.668	0.214
Week 6	12.1 ± 1.0	11.9 ± 1.4	0.240	0.239

* *p* < 0.05 is statistically significant. ^1^ Independent t-test. ^2^ One-way ANCOVA (adjusted for blood transfusion, culture proven sepsis and oxygen supplementation).

**Table 3:** Weekly mean haemoglobin levels based on the need for treatment of ROP.

Mean Haemoglobin (g/dL)				
Period	ROP not Requiring Treatment/no ROP *n* = 43	ROP Requiring Treatment *n* = 19	*p*-Value ^1^	*p*-Value ^2^
Birth	16.0 ± 2.3	14.7 ± 1.9	0.033 *	0.139
Week 1	14.6 ± 1.4	13.3 ± 1.5	0.002 *	0.219
Week 2	13.5 ± 1.2	13.2 ± 1.1	0.311	0.120
Week 3	13.4 ± 1.3	12.5 ± 0.8	0.012 *	0.139
Week 4	12.6 ± 1.0	11.9 ± 0.9	0.013 *	0.458
Week 5	12.4 ± 0.9	12.5 ± 1.6	0.790	0.771
Week 6	12.1 ± 1.1	11.9 ± 1.6	0.378	0.091

* *p* < 0.05 is statistically significant. ^1^ Independent t-test. ^2^ One-way ANCOVA (adjusted for blood transfusion, culture proven sepsis and oxygen supplementation).

**Table 4:** Summary of literature associating haemoglobin with ROP.

Name	Year	Sample Size	Country	Conclusions
Bossi et al. [30]	1984	132	Switzerland	Haemoglobin levels show no correlation with ROP.
Englert et al. [15]	2001	107	America	Anaemia is not an independent risk factor for severity of ROP.
Banerjee et al. [14]	2015	920	United Kingdom	Low haemoglobin at birth is associated with ROP in premature infants. Duration of anaemia during the first week of life is associated with an increased risk of developing ROP warranting treatment.
Lundgren et al. [17]	2018	227	Australia	Mean haemoglobin levels during the first week of life did not differ significantly between infants with ROP requiring treatment and controls after adjusting for confounders.
Lundgren et al. [18]	2019	78	Sweden	Anaemia during the first week of postnatal life is a significant risk factor for severe ROP.
Akyüz Ünsal et al. [31]	2020	142	Turkey	Haemoglobin levels in the first month of life are significantly lower in infants with ROP than those without ROP.

## Data Availability

The data presented in this study are available on request from the corresponding author.
